# A Pooled Sample Study of Opioid Use Disorder Treatment Wait Time Among a Pregnant Population in New York

**DOI:** 10.3390/epidemiologia6040065

**Published:** 2025-10-16

**Authors:** Stanley Nkemjika, Gulshan Begum, Bolaji Yoade, Vaibhav Vyas, Henry Onyeaka, Olubusola Olatunji, Olaniyi Olayinka, Ayana Jordan

**Affiliations:** 1 Department of Psychiatry and Human Behavior, Thomas Jefferson University, Philadelphia, PA 19107, USA; 2 Department of Psychiatry, Zucher Hill, Forest Hill, New York, NY 11375, USA; 3 Department of Psychiatry, Interfaith Medical Center, Brooklyn, NY 11213, USA; 4 Department of Psychiatry, Harvard Medical School, Boston, MA 02115, USA; 5 College of Health & Human Services, Northern Kentucky University, Highland Heights, KY 41099, USA; 6 Department of Psychiatry, University of Texas Health Science Center, Houston, TX 78229, USA; 7 Department of Psychiatry, NYU Grossman School of Medicine, New York, NY 10016, USA

**Keywords:** opioid use disorder, pregnancy, sociodemographic, barriers, treatment

## Abstract

**Background and Aim:** Opioid use disorder (OUD) during pregnancy has become a major public health issue, with its prevalence rising significantly in recent years. The incidence of neonatal abstinence syndrome (NAS) has also surged, from 1.5 cases per 1000 hospital births in 1999 to 6.0 cases per 1000 in 2013. This study aims to identify and analyze the concerns faced by pregnant people in accessing OUD treatment on time, specifically focusing on New York. **Methods:** The pooled sample of 225,275 individuals represents pregnant patients with OUD who received treatment at substance use disorder (SUD) facilities across New York State between 2016 and 2020, using data from the TEDS-D database. This dataset includes all pregnant individuals diagnosed with OUD, with consistent criteria for treatment eligibility applied. **Results:** The adjusted odds ratio (AOR) for medication-assisted treatment (MAT) for OUD was 1.41 (95% CI 1.15, 1.72; *p* = 0.0008) for full-time employees and 1.11 (95% CI 0.91, 1.34; *p* = 0.32) for part-time employees, compared to unemployed individuals. Regarding marital status, the AOR for treatment access was 1.51 (95% CI 1.34, 1.70; *p* < 0.0001) for currently married individuals and 1.85 (95% CI 1.67, 2.06; *p* < 0.0001) for those who are divorced or widowed, compared to individuals who have never married. **Discussion:** Our study highlights key sociodemographic barriers that affect early access to care for pregnant individuals in New York. OUD continues to be a critical public health issue, particularly among pregnant people, who are exposed to heightened health risks for both themselves and their babies, due to societal perceived stigma related to use during pregnancy.

## 1. Introduction

Opioid use disorder (OUD) during pregnancy represents a significant public health challenge, with a marked increase in its incidence over the past few decades. Diagnostic and Statistical Manual of Mental Disorders, Fifth Edition (DSM-5) has replaced the terms “opioid abuse” and “opioid dependence” with “opioid use disorder,” which is characterized by a spectrum of severity based on the presence of specific symptoms [1,2]. This shift in terminology suggests that this is a chronic, yet treatable condition that warrants comprehensive care, particularly among pregnant persons [3,4]. Even though medication for opioid use disorder (MOUD) with buprenorphine and methadone has proven effective, pregnant persons frequently encounter barriers trying to access these essential treatments, resulting in significant disparities in care [5]. The rise in opioid use during pregnancy is a growing concern [6], with the number of cases of neonatal abstinence syndrome (NAS) increasing from 1.5 cases per 1000 hospital births in 1999 to 6.0 cases per 1000 by 2013 [7]. Similarly, newer trends depict increased cases of 8.0 per 1000 hospital births based on 2016 data [8]. While this increase may reflect improvements in diagnostic awareness and medical reporting, the associated financial burden on the healthcare system continues to be a significant concern [9]. This sharp increase has significant financial implications, leading to healthcare costs of around $1.5 billion annually [8]. Further, reviews of maternal mortality reveal that substance use is a severe risk factor for pregnancy-related deaths, highlighting the urgent need to focus on effective treatment for OUD in this vulnerable group [10].

Two primary treatment modalities for pregnant people with OUD are opioid agonist therapy using buprenorphine or methadone and medical withdrawal management (detoxification) [11]. Buprenorphine acts as a partial agonist at the mu-opioid receptors, offering a lower risk of overdose and fewer drug interactions than methadone, which is a full mu agonist [12]. Both medications have been shown to improve pregnancy outcomes by reducing the risks associated with withdrawal and relapse, including fetal growth restriction, abruptio placenta, and preterm birth [4]. Despite the known benefits, early access to MOUD remains inconsistent. Research shows that many pregnant people face significant challenges when trying to access treatment for OUD. A recent study found that only a small number of providers offered buprenorphine care specifically for pregnant people [13]. Many pregnant persons must make multiple calls and often wait over a week to get an appointment [14]. While opioid treatment programs (OTPs) tend to be more welcoming to new pregnant patients, these programs are often limited in number and primarily located in urban areas, leaving many women in rural settings with few options [15].

Wait times have been consistently identified as a significant barrier to timely access of treatment for individuals with OUD [16,17,18]. Notably, delays in initiating care can lead to worsened health outcomes, continued substance use, and increased risk of overdose, particularly in vulnerable populations [19]. Among the people with OUD, extended wait times have been associated with reduced motivation to engage in treatment, heightened psychosocial distress, and a higher likelihood of treatment attrition [20]. These concerns are especially critical for pregnant individuals with OUD, for whom timely early access to care is not only a matter of personal health but also a determinant of neonatal outcomes [21]. The unique physiological, psychological, and social circumstances of pregnancy intensify the urgency of treatment, yet the extent to which wait times specifically affect this subgroup remains underexplored in the literature. Factors such as limited availability of providers trained in both addiction medicine and prenatal care, compounded by systemic barriers like insurance coverage and geographic disparities, may exacerbate delays [20]. Moreover, the stigma around substance use in pregnancy likely deters patients from continuing to engage in care when dealing with practical barriers [22]. Dealing with wait times in this context will demand an understanding that is intersectional, not only of the infrastructure for drug-recovery services [17], but also of the delivery of maternal care. Given the time-sensitive nature of pregnancy and the well-documented benefits of early treatment engagement, investigating wait times as a barrier in this context is both timely and imperative.

Timely access to MOUD is particularly critical for pregnant individuals due to the dual health risks posed to both the mother and fetus. Initiating MOUD as early as possible in pregnancy, especially in the first trimester is associated with the best outcomes for both the birthing person and the fetus [23]. Delays in receiving MOUD during pregnancy are associated with increased risks of adverse maternal and neonatal outcomes, including continued illicit opioid use, preterm birth, low birth weight, and neonatal abstinence syndrome [24,25]. While MOUD is the gold standard for treating OUD in pregnancy, systemic barriers such as limited provider availability, stigma, and restrictive treatment protocols contribute to prolonged wait times for this population [26]. Assessing whether wait times are associated with actual MOUD receipt among pregnant individuals is essential to inform service delivery models that prioritize timely intervention, reduce perinatal morbidity, and support maternal recovery trajectories. Furthermore, pregnant individuals may face more scrutiny and legal consequences related to substance use, such that it may affect their treatment-seeking behaviors and increase vulnerability during waiting periods [27]. Policies promoting immediate or same-day access to MOUD during pregnancy have shown promise in enhancing treatment initiation and improving outcomes [23,28], yet evidence remains limited on how wait times influence receipt of care in this group. Investigation of this relationship can highlight structural inequities in addiction treatment services which may lead to the implementation of low-threshold, trauma-informed approaches for pregnant populations. Thus, examining the association between wait times and MOUD receipt during pregnancy is both a clinical and public health imperative aimed at addressing a high-risk, underserved population.

Moreover, financial constraints exacerbate treatment disparities, as many clinics do not accept insurance, leading to high out-of-pocket patient costs [29,30]. This economic burden disproportionately affects marginalized populations, including women from racial and ethnic minoritized backgrounds and those in low-income brackets [31,32]. Cultural and attitudinal barriers also contribute to treatment avoidance, particularly among Latina women, who reported feeling a lack of social support for pursuing treatment [33].

Thus far, there remains poor literature on the disparate access of treatment resources in terms of factors associated with wait times among this vulnerable population. Addressing these disparities is imperative for enhancing public health outcomes, supporting maternal mental health, and improving the overall well-being of families affected by OUD. Hence, we aim to identify and analyze the factors associated with wait times faced by pregnant women in timely access of treatment for OUD, with a particular focus on New York.

## 2. Methods

### 2.1. Data and Samples

This analysis involved the Treatment Episode Data Set-Discharge (TEDS-D), an annual national compilation of records of admissions and discharges from the substance use treatment system. The TEDS system acts as a thorough database that states in the US use to keep track of their substance use treatment systems. We specifically extracted data on OUD among pregnant persons from TEDS-D, a national database managed by Substance Abuse and Mental Health Services Administration (SAMHSA) that tracks admissions and discharges from SUD treatment facilities. Data is gathered annually from all 50 states, including US territories, and covers regions such as the Northeast, Midwest, South, and West. The information is collected from the state administrative records and standardized to maintain consistency across all states. Data from this is submitted to the federal government each year, and it is estimated to represent the approximately 83% of all eligible alcohol and other drug treatment admissions in the US. In the OUD sample, an observation corresponds to a treatment episode and associated discharge records. The TEDS-D dataset includes: (1) demographic details; (2) information on primary, secondary, and tertiary substances used; (3) routes of administration; (4) usage frequency; (5) age of first use; (6) referral sources; (7) prior treatment episodes; and (8) service types, including planned use of medication for addiction treatment (MAT)—such as methadone, buprenorphine, or naltrexone. Psychiatric, social, and economic data is also collected. Additionally, TEDs capture the type of service at discharge, length of stay, and reasons for discharge or service discontinuation. In this study, we focused on pooled sample records from 2016 to 2020 involving pregnant persons aged 12 and older in New York State, collecting critical information on admission socio-demographics and characteristics of OUD (including opioid abuse, dependence—as defined by DSM IV, intravenous drug use, and the use of MOUD).

### 2.2. Analytical Sample and Inclusion Criteria

The pooled sample (N = 225,275) is representative of pregnant clients who received SUD treatment services within New York state as found by TEDS-D database From 2016 to 2020. The dataset was extracted from all pregnant patients who provided response to OUD diagnosis status, and the treatment type accepted for treatment acceptance principle were consistent. In addition, we used the data from the first acceptance at baseline (first time entering OUD treatment) to explore factors correlating with treatment on admission and waiting time. Therefore, analyses included all pregnant persons according to their diagnosis, treated in any of the healthcare facilities, and reported to the TEDS-D that were 12 years or older and reporting for treatment of SUD.

### 2.3. Measures

Dependent variable: The dependent variable is “MAT for opioid treatment”. This includes data on the use of drugs such as Methadone, Buprenorphine and Naltrexone. The answers were “yes” or “no.”

Independent Variable: The primary independent variable of concern is barriers to access measured as “days of wait time.” This is representative of the number of days waiting for treatment from the first point of contact or request for service to when the client was admitted and/or the first clinical service provided to clients. Time delays caused by not working with the client (client not available, need time to meet some requirement or obligation) are not included. Response was classified in the dataset as “0,” “1,” “2,” “3,” or “4” which corresponded with responses of “0 days,” “1 to 7 days,” “8 to 14 days,” “15 to 30 days” and “31 or more days,” respectively. Because of the few number of responses in the other cells after subgroup analysis, we further re-categorized it as “0 days,” “1–7 days,” and “>7 days.”

Confounding variable: We recorded the individual demographics, which includes the client age, biological sex, marital status, and education (years in school). Respondents also reported on psychosocial characteristics, including employment status (full-time, part-time, unemployed and seeking employment, unemployed and not seeking employment, or not in the labor force); homelessness status (stable housing or homeless); Racial and ethnic disposition was measured in five categories: Alaskan Native, Black/African American, Asian/Pacific Islander, White, and Other (includes American Indian, Asian, Other single race, two or more races and Native Hawaiian, or Other Pacific Islanders). The rest of the covariates were: length of stay, intravenous drug use, geographic region of treatment facilities, the primary expected source of payment for treatment at the time of admission, principal source of support and type of treatment settings, including detoxification, rehabilitation and ambulatory setting.

### 2.4. Statistical Analysis

Analysis includes all types of OUD medication treatment and admissions. Data on a continuous scale were expressed using mean, standard deviation, range, and median. Categorical data were described in terms of counts and percentages. Statistically significant estimates allowed us to utilize the appropriate Chi-square (χ2) tests to assess relationships and differences between groups. Multivariate logistic regression models were developed to test the association between explanatory variables (wait times and sociodemographic attributes) and MAT for OUD. Thus, multivariate logistic regression models were applied to evaluate the effect of predictive variables statistically significantly associated (*p* < 0.5) to MAT. Confounding factors were defined as those whose odds ratio changed ≥ 10% and that were included in the final logistic regression model. We also controlled for confounders by adding covariates based on literature evidence. The TEDS-D survey design did not consider weighted variables and the possibility of missing values in some variables from respondents. Hence, maintaining the statistical rigor was paramount, and unwanted observations in person-level files were not deleted in the analysis. All analyses and graphical presentations were conducted using the SAS statistical software version 9.4 suite.

### 2.5. Ethical Consideration

TEDS data are publicly available without participant identification as the study has an Institutional Review Board exempt status.

## 3. Results

### 3.1. Demographic Attributes of the Pregnant Population in New York State

The demographic characteristics of the study participants are reported on Table 1. A large proportion of the participants were aged 25 to 49 years (78.82%), followed by the age group 12–24 years (20.08%), with those greater than 50 years old constituting only 1.11% of the population (Table 1). Most of the study population identified as White (74.98%), followed by Black/African American (15.25%). A small percentage identified as Alaskan Native/American Indian (3.88%) and Asian/Pacific Islander/Hawaiian Native (0.65%). In terms of education, 41.75%, 31.32%, 18.42%, 5.84%, and 2.66% of the population had grade 12, grade 9–11, 1–3 years of college, less than 1 school grade to 8 grade, and greater 4 years of college, respectively (Table 1). Most of the population falls in the Unemployed (45.47%) and the Not in labor force (41.98%) categories, while those working Part-time and Full-time constitute 6.30% and 6.25% of the population, respectively. 71.05% of the population are Never Married, 15.49% are Divorced/Separated/Widowed, while 13.46% are currently Married.

### 3.2. Attributes Based on Opioid Use Disorder

Table 2 presents the characteristics of pregnant patients with opioid use. Regarding age, the percentages of pregnant people using opioid compared to those who did not are as follows: ages 12 to 24, 17.9% vs. 20.74%; ages 25 to 49, 81.26% vs. 78.04%; and over 50 years, 0.76% vs. 1.22%. Regarding race, the data shows Alaska Native individuals at 2.5% vs. 4.31%, Black individuals at 7.56% vs. 17.74%, and White individuals at 84.72% vs. 71.82%. Education levels showed that less than 8th grade was 5.53% vs. 5.94%, grades 9 to 11 were 25.73% vs. 33.1%, one to three years of college were 21.31% vs. 17.50%, and over four years of college were 2.90% vs. 2.59%. For primary payment sources, self-pay was 4.76% vs. 4.22%, Medicare 0.72% vs. 0.40%, and Medicaid 58.36% vs. 56.46%. In the past 30 days before admission, arrests were noted with 89.66% vs. 91.57% having no arrests, one arrest at 9.09% vs. 7.4%, and two or more arrests at 1.25% vs. 1.03%. Regarding detox admissions, inpatient or residential detox was 7.46% vs. 4.11%, rehab (both short and long-term) was 23.42% vs. 27.71%, and ambulatory intensive outpatient was 69.12% vs. 68.18%. Early access to opioid treatment showed 52.49% vs. 10.10% had access, while 47.51% vs. 89.90% did not. Lastly, for drug use, 81.11% vs. 25.47% reported using drugs, and 18.89% vs. 74.18% did not use intravenous drugs.

### 3.3. Association Based on MOUD Access and Sociodemographic Attributes

The adjusted odds (AOR) of MAT for OUD is 0.88 (95% CI 0.80, 0.96; *p* = 0.0046) and 0. 48 (95% CI 0.30, 0.79; *p* = 0.0035) in those 25–49 years and >50 years, respectively, when compared to those 12–24 years old. In terms of race, when compared to Black/African American, the AOR of MAT for OUD in Alaskan Native/American Indian, Asian/Pacific Islander/Hawaiian Native, and White are 3.81 (95% CI 2.70, 5.39; *p* < 0.0001), 2.50 (95% CI 1.57, 3.96; *p* < 0.0001), and 0.97 (95% CI 0.84, 1.11; *p* = 0.6057) (Table 3). Those who achieved Grade 9–11, Grade 12, 1–3 years of college, and greater than 4 years of college had an AOR of MAT for OUD of 1.13 (95% CI 0.95, 1.34; *p* = 0.1764), 0.89 (95% CI 0.75, 1.04; *p* = 0.1394), 0.83 (95% CI 0.70, 0.99; *p* = 0.0395), and 0.71 (95% CI 0.54, 0.92; *p* = 0.0101), respectively, when compared with those who achieved less than 1 school grade–8 grade. The AOR of MAT for OUD is 1.41 (95% CI 1.15, 1.72; *p* = 0.0008), and 1.11 (95% CI 0.91, 1.34; *p* = 0.32) in full-term and part-time employee, respectively, when compared with the unemployed. In terms of the marital status, the AOR of MAT for OUD was 1.51 (95% CI 1.34, 1.70; *p* < 0.0001), and 1.85 (95% CI 1.67, 2.06; *p* < 0.0001) in those who are currently married and divorced/widowed, respectively, when compared to those who never married. When compared to the homeless, those who live dependently and independently have a AOR of MAT for OUD of 0.70 (95% CI 0.62, 0.79; *p* < 0.0001), and 0.77 (95% CI 0.69, 0.86; *p* < 0.0001). The AOR of MAT for OUD are 0.92 (95% CI 0.77, 1.10; *p* = 0.3737), 1.23 (95% CI 0.96, 1.57; *p* = 0.1069), and 1.01 (95% CI 0.86, 1.18; *p* = 0.9133) in those on public assistance, Retirement/Pension/Disability, and those with no source of income, respectively, when compared with those on wages/salary (See Table 3).”

## 4. Discussion

OUD remains a severe public health burden, especially among vulnerable groups like pregnant persons who are facing unique challenges such as increased health risks for both themselves and their babies, limited early access to specialized care, and the stigma surrounding substance use during pregnancy [34,35]. Early access to MOUD remains a critical issue, as our study reveals a stark disparity in treatment access. Among those pregnant persons exposed to opioids, a little over half (52.49%) had accessed treatment, while 47.51% did not access treatment. The significant barriers to timely access of treatment underscore the urgent need for policies aimed at improving the availability and accessibility of OUD treatment services for pregnant persons. For example, our study showed significant disparities in the types of admissions for OUD treatment among pregnant people in New York State. Specifically, AOR for medical withdrawal management (detoxification) admissions compared to ambulatory treatment was 0.386, indicating that pregnant persons were less likely to be admitted to medical withdrawal management facilities than to receive outpatient care. On the other hand, the AOR for rehabilitation admissions in comparison to ambulatory treatment was 1.136, indicating a slight tendency or increased likelihood for these people to be directed toward inpatient rehabilitation services instead of outpatient care. This distinction highlights the complexities of treatment pathways for pregnant persons, emphasizing the need for integrated care approaches that can better cater to their specific circumstances and vulnerabilities.

Our analysis demonstrates that these specific needs include limited early access to healthcare, stigma and discrimination, lack of comprehensive care, social determinants of health, limited early access to MOUD, cultural and language barriers and mental health challenges [36,37,38]. Early access to MOUD remains a critical issue, as our study reveals a stark disparity in treatment access. Waiting times for opioid treatment present further insights into access disparities. Pregnant persons waiting 1–7 days for treatment had an AOR of 0.786, indicating that shorter wait times were associated with higher odds of receiving treatment, which is consistent with existing literature that emphasizes the negative impact of delays on treatment engagement [39]. For instance, the literature evidence indicated that pregnant persons paying cash at an opioid treatment program (OTP) experienced a predicted wait time of just 1.3 days, compared to 16.7 days for those with insurance at a buprenorphine provider [15]. Likewise, pregnant persons paying cash at an OTP had an even shorter wait time of 0.8 days, while those with insurance faced a significantly longer predicted wait time of 6.7 days, highlighting the disparities in early access to treatment based on payment methods [15]. In contrast, those waiting longer than a week had an AOR of 1.107, suggesting that extended waiting periods may not significantly influence treatment access but could still contribute to a sense of urgency among pregnant patients. These findings portray the critical need for timely early access to care and highlight the importance of addressing systemic factors associated with wait times that may disproportionately affect pregnant persons seeking OUD treatment [40]. The significant barriers to timely access of treatment highlight the urgent need for policies aimed at improving the availability and accessibility of OUD treatment services for pregnant people.

Sociodemographic findings from our study demonstrate that living arrangements significantly affect the likelihood of opioid use and treatment access. Pregnant persons living independently (0.77, *p* < 0.0001) or dependently (0.70, *p* < 0.0001) have lower odds of barriers assessing treatment for opioid use compared to those who are homeless. This suggests that stable housing conditions may contribute to better health outcomes, indicating a need for programs that provide housing support alongside treatment services. Our analysis also reveal that younger pregnant persons (ages 12 to 24) have a higher prevalence of opioid use (20.74%) compared to older age groups (17.9% for pregnant people using opioids). Conversely, the likelihood of opioid use increases significantly among pregnant persons aged 25 to 49 (81.26% opioid users vs. 78.04% non-users) and decreases for those over 50 years (0.76% vs. 1.22%). Our study showed that opioid use treatment access in pregnancy has a statistically significant association with age. The AOR indicates that pregnant persons aged 25 to 49 and over 50 have lower odds of timely access of treatment compared to younger pregnant people, with AORs of 0.88 and 0.48, respectively. This suggests that younger pregnant persons may be more vulnerable to opioid use, necessitating targeted interventions that address the unique challenges faced by this age group. Our results are consistent with other studies in the literature, which demonstrates that older pregnant persons with OUD frequently encounter additional obstacles to treatment, which causes major delays in receiving therapy. On the other hand, pregnant people who began using opioids earlier in life typically have less concerns and have faster early access to treatment programs [39]. It is worth noting, however that this data does not support the study conducted by Nguyen et al. (2023) who reported lower odds of treatment access among pregnant persons using opioids who are less than 20 years old, although not statistically significant [41]. Hence, warrants further exploration in future studies.

Racial and ethnic differences were prominent in this study. We noted that Alaska Native/American Indian and Asian/Pacific Islander pregnant persons have significantly higher odds of timely access of treatment compared to Black pregnant persons, with AORs of 3.81 and 2.50, respectively. These findings highlight the importance of culturally sensitive treatment programs that acknowledge and address the specific needs of racially and ethnically diverse populations. Although our study shows that White pregnant people are 0.13 times less likely to experience treatment barriers when compared to the Black/African American, this was not statistically significant. Education is another critical factor influencing opioid use and treatment access. Notably, the likelihood of opioid use decreases with higher educational attainment, with those holding less than 8th-grade education showing a higher prevalence. AORs for timely access of treatment indicate that women with one to three years of college (0.83, *p* = 0.0395) and more than four years of college (0.71, *p* = 0.0101) were less likely to experience barrier to access treatment than those with less than 8th-grade education. Thus, women with higher education levels might be more aware of treatment options and available resources. Hence, it emphasizes the need for educational initiatives to improve early access to care and knowledge for those with lower educational backgrounds. Additionally, our study showed that employment status may be influential towards treatment access among pregnant persons with OUD, as 44.28% of unemployed pregnant persons had a higher likelihood of opioid use. The AOR for treatment access revealed that full-time employed women were more likely to access treatment (1.41, *p* = 0.0008) than the unemployed. Similarly, being used part-time exhibited a weaker effect (1.11, *p* = 0.32), which was not statistically significant. These findings suggest that having stable employment might serve as a protective factor, facilitating better early access to healthcare resources, including treatment for opioid use disorder (OUD). In terms of marital status, pregnant persons who were currently married (1.51, *p* < 0.0001) or divorced/widowed (1.85, *p* < 0.0001) were more likely to access treatment compared to those who have never been married. Thus, social support networks and relationship stability can enhance the probability of seeking treatment, emphasizing the importance of incorporating family and partner support in intervention strategies.

The stigma around substance use and beliefs about the effectiveness of treatment, play a significant role in maintaining disparities in care [15]. For instance, Latina and Black women often face more negative attitudes toward treatment than their White counterparts, which can seriously impact their willingness to seek help [42,43]. These insights portray the urgent need for culturally sensitive care and targeted support that addresses unique challenges faced in diverse communities [44]. The implications of inadequate availability to culturally informed care extends far beyond individual health. Considering OUD remains untreated during pregnancy, it not only jeopardizes the health of pregnant persons, but also affects fetal development and newborn well-being. Notably, there is a relationship between untreated SUDs and inadequate prenatal care, leading to higher rates of complications like preterm birth and low birth weight [45]. This situation strains healthcare resources [46,47]. Therefore, tackling these disparities is essential for enhancing the health outcomes of both pregnant people and infants, and for reducing the long-term societal costs associated with untreated SUDs. Additionally, the stigma surrounding OUD and treatment must be actively challenged through public education campaigns and community engagement initiatives [48]. These efforts should aim to normalize the conversation around use disorder and highlight the importance of seeking treatment. Such initiatives could contribute to a more supportive environment for pregnant client with OUD, encouraging them to pursue necessary care without fear of judgment or discrimination.

This study has several strengths that contribute to our understanding of this critical public health issue. One significant strength is its focus on a vulnerable population, which allows for a detailed analysis of the unique challenges faced by pregnant persons seeking treatment. Hence, by utilizing a large, diverse sample, the study provides robust insights into the demographic, socioeconomic, and treatment-related factors influencing early access to care. However, the study also has limitations that must be acknowledged. Due to its cross-sectional design, it restricts the ability to establish causal relationships between the identified disparities and treatment outcomes. Additionally, this study is prone to self-reported bias, as participants might underreport or overreport their drug use and treatment experiences due to stigma or recall inaccuracies. Furthermore, the study may not account for all confounding variables that could influence treatment access, such as geographic barriers, personal health conditions, or the availability of local resources, which can vary significantly across regions. Despite these limitations, the study serves as a crucial step toward addressing opioid use disorder among pregnant persons in New York.

## 5. Conclusions

In conclusion, our study reveals significant sociodemographic challenges and factors associated with wait times that may impact early access to care among pregnant persons in New York. While stable employment and education levels may enhance treatment awareness and access, many pregnant people still face obstacles, particularly regarding the types of treatment available and waiting times for OUD treatment services. The lower likelihood of medical withdrawal management admissions compared to outpatient care suggests a need for more comprehensive treatment options tailored to the unique needs of pregnant persons, as detoxification is not treatment. Furthermore, the urgency of timely early access to treatment must be addressed, as longer waiting periods to access services can deter engagement in necessary care. Hence, ensuring culturally sensitive health policies is crucial for improving health outcomes for both pregnant persons and their children.

## Figures and Tables

**Table 1:** Sociodemographic Descriptive of Pregnant New York Sample with OUD Response (N = 225,275).

Demographic Characteristics	Frequency	Percentage
Age		
12–24	45,231	20.08
25–49	177,551	78.82
>50	2493	1.11
Race		
Alaskan Native/American Indian	8197	3.88
Asian/Pacific Islander/Hawaiian Native	1377	0.65
Black/African American	32,195	15.25
White	158,348	74.98
Other single race/two or more races	11,064	5.24
Education		
<1 school grade–8 grade	12,839	5.84
Grade 9–11	68,863	31.32
Grade 12	91,780	41.75
1–3 years of college	40,495	18.42
>4 years	5858	2.66
Employment status		
Full-time	13,878	6.25
Part time	13,983	6.30
Unemployed	100,970	45.47
Not in labor force	93,221	41.98
Marital Status		
Never married	121,542	71.05
Currently married	23,026	13.46
Divorced/separated/widowed/	26,497	15.49
Health Insurance		
Private	4912	4.80
Medicaid	60,725	59.36
Medicare, other (TRICARE, etc.)	6827	6.67
None	29,830	29.16
Services waiting time		
Zero days	77,968	65.09
1–7 days	18,589	15.52
>1 week	23,225	19.39
Arrest in past 30 days prior to admission		
None	199,756	91.12
Once	17,111	7.80
Two or more times	2368	1.08
Source of income		
Wages/salary	20,396	15.89
Public assistance	21,806	16.99
Retirement/pension, disability	5108	3.98
Other	11,117	8.66
None	28,259	22.01
Living arrangement		
Homeless	31,337	14.18
Dependent living	52,197	23.62
Independent living	137,440	62.20
Type of treatment Service		
Detox, (hospital inpatient, residential)	5726	5.10
Rehab, (both short and long term)	29,673	26.44
Ambulatory, Intensive outpatient	76,816	68.45
Primary Payment Source		
Self-Pay	3745	4.35
Private insurance	2129	2.47
Medicare	410	0.48
Medicaid	49,038	56.91
Other government payments	24,548	28.49
No charge (free, charity, teaching, etc.)	819	0.95
Other	2744	3.18
Missing/unknown not collected/invalid	1758	2.04

**Table 2:** Sociodemographic Characteristics vs. Opioid Use Disorder in Pregnancy.

Demographic Characteristics	Opioid Use		Total
	No%	Yes%	
Age			225,275
12–24	20.74	17.98	
25–49	78.04	81.26	
>50	1.22	0.76	
Race			211,181
Alaskan Native/American Indian	4.31	2.56	
Asian/Pacific Islander/Hawaiian Native	0.72	0.45	
Black/African American	17.74	7.56	
White	71.82	84.72	
Other single race/two or more races	5.41	4.70	
Employment			222,052
Full-time	6.84	4.38	
Part time	6.67	5.11	
Unemployed	45.85	44.28	
Not in labor force	40.63	46.22	
Marital status			171,065
Never married	69.42	75.85	
Currently married	13.94	12.04	
Divorced/separated/widowed/	16.63	12.12	
Education			219,835
<1 school grade–8 grade	5.94	5.53	
Grade 9–11	33.10	25.73	
Grade 12	40.87	44.53	
1–3 years of college	17.50	21.31	
>4 years	2.59	2.90	
Primary Payment Source			8617
Self-Pay	4.22	4.76	
Private insurance	2.40	2.70	
Medicare	0.40	0.72	
Medicaid	56.46	58.36	
Other government payments	28.99	26.85	
No charge (free, charity, teaching, etc.)	0.95	0.96	
Other	3.20	3.14	
Missing/unknown not collected/invalid	1.27	0.70	
Health Insurance			102,294
Private	4.44	5.97	
Medicaid	58.91	60.81	
Medicare, other (TRICARE, etc.)	6.87	6.05	
None	29.78	27.17	
Primary Source of Income			84,413
Wages/salary	17.23	11.85	
Public assistance	17.22	16.29	
Retirement/pension, disability	4.16	3.43	
Other	7.59	11.90	
None	19.11	30.79	
Living Arrangements			220,974
Homeless	14.23	14.04	
Dependent living	24.04	22.29	
Independent living	61.73	63.66	
Arrest In past 30 days Prior to Admission			219,235
None	91.57	89.66	
Once	7.40	9.09	
Two or more times	1.03	1.25	
Detox			112,215
Detox, (hospital inpatient, residential)	4.11	7.46	
Rehab, (Both short and long term)	27.71	23.42	
Ambulatory, Intensive outpatient	68.18	69.12	
Medication-Assisted Opioid Therapy			216,411
Yes	10.10	52.49	
No	89.90	47.51	
IV Drug Use			153,822
Yes	25.47	81.11	
No	74.18	18.89	
Other Opiates/Synthetics			225,275
Yes	25.92	15.78	
No	74.08	84.22	
Heroin			225,275
Yes	5.75	100.00	
No	94.25	0.00	
ADHD			225,275
Yes	0.01	0.00	
No	99.99	100.00	
Bipolar			225,275
Yes	0.25	0.12	
No	99.75	99.88	

**Table 3:** Adjusted Odds Ratio for Opioid Use Disorder Treatment Barriers among Pregnant Population.

Dependent Variables	Adjusted Odds Ratio	95% CI	*p* Value
Age			
12–24	ref		
25–49	0.879	0.804–0.961	0.0046
>50	0.484	0.297–0.788	0.0035
Race			
Alaskan Native/American Indian	3.813	2.698–5.387	<0.0001
Asian/Pacific Islander/Hawaiian Native	2.496	1.573–3.962	0.0001
Black/African American	ref		
White	0.965	0.842–1.105	0.6057
Other single race/two or more races	0.748	0.588–0.950	0.0175
Education			
<1 school grade–8 grade	ref		
Grade 9–11	1.126	0.948–1.338	0.1764
Grade 12	0.885	0.752–1.041	0.1394
1–3 years of college	0.832	0.698–0.991	0.0395
>4 years	0.708	0.544–0.921	0.0101
Employment status			
Full-time	1.410	1.153–1.725	0.0008
Part-time	1.105	0.909–1.343	0.3160
Unemployed	ref		
Not in the labor force	0.902		0.0134
Marital Status			
Never married	ref		
Currently married	1.505	1.335–1.696	<0.0001
Divorced/separated/widowed/	1.854	1.667–2.061	<0.0001
Living Arrangement			
Homeless	ref		
Dependent living	0.701	0.619–0.793	<0.0001
Independent living	0.771	0.692–0.860	<0.0001
Source of income			
Wages/salary	ref		
Public assistance	0.921	0.768–1.104	0.3737
Retirement/pension, disability	1.227	0.957–1.573	0.1069
Other	0.930	0.779–1.110	0.4207
None	1.009	0.860–1.183	0.9133
Type of treatment Service			
Detox, (hospital inpatient, residential)	0.386	0.329–0.454	<0.0001
Rehab, (Both short and long term)	1.136	1.036–1.245	0.0068
Ambulatory, Intensive outpatient	ref		
Services waiting time			
Zero days	ref		
1–7 days	0.786	0.717–0.861	<0.0001
>1 week	1.107	0.974–1.258	0.1187

## Data Availability

https://www.samhsa.gov/data/data-we-collect/teds-treatment-episode-data-set (10 September 2024).
